# Clinical practice guideline adaptation for risk-based caries management in 18–55 year-old Iranian adults

**DOI:** 10.1186/s12903-022-02699-w

**Published:** 2023-01-06

**Authors:** A. Pakdaman, N. Gholizadeh, M. J. Kharazifard, M. Eshrati

**Affiliations:** 1 Research Center for Caries Prevention, Dentistry Research Institute, Tehran University of Medical Sciences, Tehran, Iran; 2 Community Oral Health Department, School of Dentistry, Tehran University of Medical Sciences, Tehran, Iran; 3 Oral and Maxillofacial Medicine Department, School of Dentistry, Tehran University of Medical Sciences, Tehran, Iran; 4 Dental Research Center, Dentistry Research Institute, Tehran University of Medical Sciences, Tehran, Iran

**Keywords:** Dental caries, Risk assessment, Preventive dentistry, Practice guideline, Adaptation

## Abstract

**Purpose:**

To adapt an evidence-based clinical practice guideline (CPG) for risk-based management of caries in 18–55 year-old Iranian adults.

**Methods:**

A multidisciplinary adaptation team reviewed evidence-based guidelines such as the NICE, SIGN, and ADA according to the defined clinical questions. In addition, databases such as the PubMed and Google Scholar were searched and CPGs were screened and appraised using the AGREE II (Appraisal of Guidelines for Research and Evaluation II) tool. Clinical scenarios were developed and their level of evidence, clinical advantage and adaptability were assessed. Following a two-round ranking by experts, the final recommendations were selected using the RAND-UCLA appropriateness method.

**Results:**

Of 17 CPGs, 5 were selected as the source guidelines for adaptation. To assess the risk of caries in the adult population, reduced Cariogram (without saliva tests) and CAMBRA were suggested as diagnostic tools. In addition, 53 risk-based recommendations on the preventive care (including the use of fluoride toothpaste, fluoride, and chlorhexidine mouthwash, at home and in-office fluoride gel, fluoride varnish, mouth buffering, and sealant), operative intervention threshold, and follow-up interval were adapted for Iranian adults.

**Conclusions:**

A guideline was adapted for risk-based management of dental caries in Iranian adults. This helps local dentists in decision making and promoting oral health of adults. Further research is needed to assess the external validity and feasibility of the adapted guideline in the Iranian population.

**Supplementary Information:**

The online version contains supplementary material available at 10.1186/s12903-022-02699-w.

## Introduction

Clinical practice guidelines (CPGs) represent a series of recommendations based on the best available evidence, mainly systematic reviews, to guide healthcare providers in clinical decision-making and to reduce practice diversities. CPGs can also reduce health inequalities and health expenses [1]. Despite these advantages, available CPGs may not be widely used in developing countries due to resource limitations, health system differences, and varied prevalence and incidence rates of the target disease [2]. Considering time, financial and human resources required for developing a new local CPG, adaptation of available CPGs is considered an option [3].

Caries risk assessment (CRA) is defined as determining the probability of developing new carious lesions or progression of existing carious lesions during a certain period [4, 5]. According to the literature, certain risk factors and risk indicators influence the occurrence or progression of dental caries, such as past caries experience, enamel defects, dental biofilm, oral hygiene, diet, and socioeconomic status of the individuals [6]. Many CRA tools are now available in the form of checklists such as the CAMBRA [7] and ADA [8] or as software programs such as the Cariogram [9] and Previser [10].

The predictive validity of these tools is still under investigation, especially in different settings [11–13]. Caries risk assessment is considered essential for delivering a suitable preventive regimen and to manage a non-cavitated carious lesion properly [14, 15]. Risk-based management of carious lesions includes a combination of patient’s caries risk assessment and delivering preventive care. This approach includes oral hygiene instructions, diet counseling, dry mouth management [16], pit and fissure sealants [17], at-home use of fluoride products such as toothpaste and mouth rinses with different fluoride concentrations [18], in-office topical fluoride therapy such as application of gels or varnishes [19], use of antimicrobial agents such as chlorhexidine mouthrinse or gel [20], and use of xylitol chewing gum or lozenges [21].

The patient’s risk status, costs, culture, and availability of resources in the region must be considered in formulating a treatment plan. Therefore, the adapted CPGs are considered as one of the main sources of evidence-based clinical decision-making [3]. The prevalence of dental caries is high in Iranian adults. The mean DMFT ranges from 4.3 in 18-year-olds [22] to 13.2 in the age group of 35–44 years [23]. Besides, Iranian dentists tend to over-treat low-risk patients [24] and have insufficient knowledge and practice regarding caries risk assessment and risk management [25]. Considering the inconsistencies regarding risk-based management of caries among available guidelines, and since no CPGs are available on caries management in adults in Iran, the aim of the present study was to adapt an evidence-based CPG for risk-based caries management in 18–55 year-old Iranian adults.

## Materials and methods

Guideline adaptation steps were followed according to the Iranian Ministry of Health and Medical Education’s national model for CPG adaptation [3]. This model was proposed based on reviewing valid CPG adaptation models such as ADAPTE [26]. The study started after obtaining clearance from the National Committee of Ethics in Biomedical Research (IR.TUMS.DENTISTRY.REC.1400.092).

### Steering committee and expert panel

A multidisciplinary team of 10 experts was assembled, including three dental public health specialists, two restorative dentistry specialists, two oral disease specialists, one general dentist, two epidemiologists with previous experience of guideline adaptation (one of them was a general dentist), and an expert searcher. The panel members were selected by convenience sampling from faculty members of Tehran University of Medical Sciences. Most of them had at least five years of experience in dental practice in public and private settings and two of them were former members of the Strategy Planning Committee of the Oral Health Office of the Ministry of Health.

The steps were clearly explained to all members and they were asked to verbally declare any conflict of interest (COI) based on items mentioned in ADAPTE toolkit ver.2.0 COI disclosure sample [26]. The panel members were contacted via email and WhatsApp for individual scorings and in person for further discussion.

### Defining clinical questions

In order to prioritize the CPG scopes and clinical questions, a list of potential measures was extracted from (1) primary screening of existing guidelines on this topic and their recommendations, (2) informal interviews with general dentists and specialists, and (3) a literature review regarding Iranian dentists’ knowledge, attitude and practice. These measures were further prioritized based on importance, cost-effectiveness, availability, and being similar or controversial among different guidelines, different practitioners, and different risk groups.

Fifty-four clear and specific clinical questions were defined according to the PIPOH framework [27]. In the present study, the PIPOH framework included (P) Patient population: 18–55 year-old Iranian adults at risk of caries including low-risk, medium-risk, high-risk, and extreme-risk, (I) Intervention: preventive and non-invasive measures, (P) Professionals: general dentists, (O) Outcome: decreased caries incidence or progression rate, (H) Healthcare setting: primary and secondary dental care providers.

To assign adults in the above caries risk groups, the reduced Cariogram software (without saliva tests) or the CAMBRA form (in the absence of the reduced Cariogram software) was suggested based on a previous study [28]. The output of the Cariogram is *Actual chance to avoid new cavities* in percentage categorized as low risk (76–100%), medium-risk (51–75%), high-risk (26–50%) and extreme-risk (0–25%) [9]. The latest version of the CAMBRA risk assessment tool directly assigns the patients to the four caries risk groups from low to extreme based on its scorings [7].

### Searching for CPGs

A search was conducted in national and international websites publishing CPGs such as the NICE [29] and SIGN [30] as well as specialized dentistry websites such as the ADA [31] and CDA [32] using the following key words: dental caries, caries risk assessment, caries management, caries prevention, dental care, guideline, practical guideline, clinical practice guideline, protocol, government publication. The major search engines such as the PubMed and Google Scholar were also searched using specific search strategies (Additional file 1). The search was limited to clinical practice guidelines addressing caries management considering the caries risk published between 2000 and 2022 in English. The guidelines specific to children and adolescents were excluded. The included websites and search engines are listed in Table 1.

**Table 1:** List of websites included in searching for CPGs

International websites	URL
National Institute for Clinical Evidence (NICE)	https://www.nice.org.uk/
Scottish Intercollegiate Guidelines Network (SIGN)	https://www.sign.ac.uk/
California Dental Association (CDA)	https://www.cda.org/
American Dental Association (ADA)	https://www.ada.org/
Public Health England (PHE)	https://www.gov.uk/government/organisations/public-health-england
NHS Health Scotland	http://www.healthscotland.scot/
ICDAS Foundation	https://www.iccms-web.com/
World dental federation (FDI)	https://www.fdiworlddental.org/
Scottish Dental Clinical Effectiveness Program (SDCEP)	https://www.sdcep.org.uk/
Guidelines International Network (GIN)	https://g-i-n.net/
National Health and Medical Research Council (NHMRC)	https://www.nhmrc.gov.au/
The Alliance for the Implementation of Clinical Practice Guidelines (AiCPG)	https://aicpg.org/
New Zealand Guidelines Group	https://www.health.govt.nz/about-ministry/ministry-health-websites/new-zealand-guidelines-group
General Dental Council (GDC)	https://www.gdc-uk.org/
UK guidelines	https://www.guidelines.co.uk/

National websites	URL
Iranian Dental Association (IDA)	https://www.ida-dent.org/
Iranian General Dentists Association (IGDA)	http://en.igda.ir/
Islamic republic of Iran ministry of health and medical education (MHME)	https://behdasht.gov.ir/
Tehran University of Medical Sciences	https://tums.ac.ir/
Iran University of Medical Sciences	https://iums.ac.ir/
Shahid Beheshti university of medical sciences	https://sbmu.ac.ir/
Tabriz University of Medical Sciences	https://www.tbzmed.ac.ir/
Isfahan University of Medical Sciences	https://mui.ac.ir/fa

Search engines	URL
PubMed	https://pubmed.ncbi.nlm.nih.gov/
Google	https://www.google.com/
Google scholar	https://scholar.google.com/
Trip data base	https://www.tripdatabase.com/
Scientific Information Database	https://www.sid.ir/

A complementary search was also conducted to identify the related evidence such as recent systematic reviews, cost-effectiveness studies and local data to assist in updating and adapting the recommendations of retrieved guidelines (Additional file 1). In addition, the ADA guidelines on non-restorative treatment of caries [33], pit and fissure sealants [34], topical fluoride [35], and non-fluoride preventive agents [21] were also considered as adjunct guidelines in the adaptation process.

### Screening and appraisal of CPGs

Relevant guidelines were first screened for (1) appropriate organization, (2) currency (publication year and latest updates), and (3) availability of the full guideline including references. In the appraisal phase, quality assessment was done using the AGREE II (Appraisal of Guidelines for Research and Evaluation II) tool, which uses 23 items to assess the quality of a CPG in 6 domains including (1) scope and purpose, (2) stakeholders’ involvement, (3) rigor of development, (4) clarity of presentation, (5) applicability, and (6) editorial independence [36]. Three members of the adaptation team independently scored each item from 1 (strongly disagree) to 7 (strongly agree). The final score was calculated using the following formula (range: 0–100 for each domain):

$$\frac{sum\;of\;the\;given\;scores\;in\;each\;domain - minimum\;possible\;score\;in\;each\;domain}{{maximum\;possible\;score\;in\;each\;domain - minimum\;possible\;score\;in\;each\;domain}}*100$$

### Clinical scenarios

As described by Fitch et al. [37], clinical scenarios are defined as potential recommendations that answer clinical questions; these scenarios are accepted or rejected as final recommendations according to the expert panel scoring. Sixty-four clinical scenarios were developed by extracting PIPOH items from the existing recommendations or, if needed, from their background data and rewriting them in a desired recommendation template based on the caries risk. The clinical scenarios were assessed by the expert panel according to the national model for CPG adaptation [3] including (1) background evidence, (2) clinical advantages, and (3) adaptability. The clinical advantage of each scenario was rated considering costs, side benefits, and side effects, and their adaptability was rated based on usability, generalizability of effectiveness and acceptability from 1 (low) to 3 (high). Based on this assessment, each scenario was finally scored from 1 (completely disagree) to 9 (completely agree).

### Decision-making

To achieve a consensus, Gestalt decision-making based on the RAM model (RAND-UCLA appropriateness method) was utilized [37]. In the first round of scoring, each expert ranked scenarios individually, which was further discussed and rescored for the second round in a panel meeting. The median score was calculated for each scenario, and its appropriateness was determined as inappropriate (1–3), uncertain (4–6), or appropriate (7–9). In addition, agreement was assessed according to the RAM model based on the panel size (Additional file 2) [37].

In the first round, appropriate scenarios with total agreement were accepted. Scenarios with uncertainty or disagreement were assessed individually and proposed for further discussion in the second round of the expert panel. If there was total agreement on the inappropriateness of a scenario, it was recommended to exclude it in the first round without further discussion [37]. After redetermining *appropriateness* and *agreement* in the second round, the scenarios were prioritized. The scenarios with disagreement and those considered inappropriate were excluded. The scenarios with total and partial agreement as “appropriate” were considered as the first and second priority, respectively. Those with total and partial agreement as “uncertain” were considered as the third and fourth priority, respectively. In case of having more than one scenario for a clinical question, the scenario with the highest priority was selected as the final recommendation.

## Results

Seventeen relevant CPGs were retrieved of which eleven were excluded due to not providing specific risk-based recommendations for caries management and one due to unavailability of the full guide following preliminary screening (Additional file 3). Five were selected as source guidelines for adaptation and were further assessed by the AGREE II as shown in Table 2. CAMBRA [7], CariesCare [38], ICCMS [14] and Malaysian guideline [39] scored 60 or more in terms of development rigor and CMS [15] scored 59.7. All five CPGs scored 70 or more in other domains.

**Table 2:** Source guidelines for adaptation and their appraisal with AGREE II

	CPGs^1^ (publication year, country)				
	Malaysian guideline (2021, Malaysia) [39]	CAMBRA (2021, USA) [7]	Caries-Care (2019, UK) [38]	ICCMS (2014, UK) [14]	CMS (2008, Australia) [15]
*Domains*					
Domain 1: Scope and Purpose	96.30	88.89	98.15	98.15	96.30
Domain 2: Stakeholder Involvement	87.04	85.19	83.33	85.19	81.48
Domain 3: Rigor of Development	83.33	60.42	63.19	70.14	59.72
Domain 4: Clarity of Presentation	98.15	87.04	90.74	90.74	92.59
Domain 5: Applicability	80.56	81.94	80.56	79.17	83.33
Domain 6: Editorial Independence	94.44	91.67	88.89	88.89	88.89
*Overall guideline assessment*	83.33	88.88	72.22	83.33	77.77

^1^Clinical Practice Guidelines

The list of 54 final adapted recommendations with their priority, source CPG(s), and background evidence is presented in Table 3 and summarized in Table 4.

**Table 3:** Final recommendations and their priority after two rounds of scoring based on RAND-UCLA appropriateness method

Scope	Domain	Final recommendation (Ref.)	Priority^1^	Source guideline(s)
Diagnosis	Diagnostic tool	1. The reduced Cariogram software (without saliva tests), or the CAMBRA form (in the absence of the reduced Cariogram software) is recommended for caries risk assessment in adults [11–13, 44]	1	–
Preventive measure	Fluoride concentration of toothpaste	2. For low-risk adults, toothbrushing twice a day, using over the counter fluoride toothpastes (1000–1450 ppm F) is recommended [9, 20, 42, 44, 45, 48, 58, 68–71]	1	Malaysian
				CAMBRA
				CariesCare
				ICCMS
				CMS
		3. For medium-risk adults, toothbrushing twice a day, using over the counter fluoride toothpastes (1000–1450 ppm F) is recommended [20, 42, 44, 45, 48, 58, 68–71]	1	Malaysian
				CAMBRA
				CariesCare
				CMS
		4. For high-risk adults, toothbrushing twice a day, using high fluoride toothpastes (1450 ppm F or more) is recommended [42, 44, 45, 72–75]	1	Malaysian
				CariesCare
				ICCMS
		5. For extreme-risk adults, toothbrushing twice a day, using high fluoride toothpastes (1450 ppm F or more) is recommended [42, 44, 45, 72–75]	1	Malaysian
				CariesCare
				ICCMS
	Sodium fluoride mouthrinse	6. For low-risk adults, use of NaF mouthrinse is not recommended [9, 18, 20, 48, 58, 68, 76, 77]	1	CAMBRA
				ICCMS
				CMS
		7. For medium-risk adults, daily use of 220 ppm NaF mouthrinse or weekly use of 900 ppm NaF mouthrinse, at a time other than brushing and with 1 min duration, is recommended [9, 18, 20, 48, 58, 68, 76, 77]	1	CAMBRA
				ICCMS
				CMS
		8. For high-risk adults, daily use of 220 ppm NaF mouthrinse or weekly use of 900 ppm NaF mouthrinse, at a time other than brushing and with 1 min duration, is recommended [9, 18, 20, 48, 58, 68, 76, 77]	1	ICCMS^2^
		9. For extreme-risk adults, daily use of 220 ppm NaF mouthrinse or weekly use of 900 ppm NaF mouthrinse, at a time other than brushing and with 1 min duration, is recommended [9, 18, 20, 48, 58, 68, 76, 77]	1	ICCMS^2^
	Chlorhexidine mouthrinse	10. For low-risk adults, use of chlorhexidine mouthrinse is not recommended [20, 48, 58]	1	CAMBRA
				CMS
		11. For medium-risk adults, use of chlorhexidine mouthrinse is not recommended [20, 48, 58]	1	CAMBRA
				CMS
		12. For high-risk adults, daily use of %0.12 chlorhexidine gluconate mouthrinse for 1 week in each month, at least 1 h apart from tooth brushing and with 1 min duration, is recommended [20, 48, 58]	1	CAMBRA
		13. For extreme-risk adults, daily use of %0.12 chlorhexidine gluconate mouthrinse for 1 week in each month, at least 1 h apart from tooth brushing and with 1 min duration, is recommended [20, 32, 48, 58]	1	CAMBRA
				CMS
	Fluoride varnish	14. For low-risk adults, routine application of fluoride varnish is not recommended [20, 48, 58]	1	CAMBRA
		15. For medium-risk adults, application of fluoride varnish, every 6 months, is recommended [18, 59, 77, 78]	1	ICCMS
		16. For high-risk adults, application of fluoride varnish, every 4–6 months, is recommended [18, 20, 48, 58, 59, 77, 78]	1	CAMBRA
				ICCMS
		17. For extreme-risk adults, application of fluoride varnish, every 3–4 months, is recommended [18, 20, 48, 58, 59, 77, 78]	1	CAMBRA
				ICCMS
	In office fluoride gel^3^	18. For low-risk adults, routine application of NaF gel is not recommended [18, 50, 77]	1	ICCMS
		19. For medium-risk adults, application of %2 NaF gel at office is recommended [18, 50, 77]	2	ICCMS
		20. For high-risk adults, application of %2 NaF gel at office is recommended [18, 50, 77]	1	ICCMS
		21. For extreme-risk adults, application of % NaF gel at office is recommended [18, 50, 77]	1	ICCMS
	At home fluoride gel	22. For extreme-risk adults, in case of caries progression despite receiving toothpaste, mouthrinse and varnish, daily application of 5000 ppm fluoride gel by at-home trays, with 5-min duration, is recommended [20, 48, 58]	1	CAMBRA
	Mouth buffering	23. For extreme-risk adults, ad libitum rinsing of the mouth with water and baking soda (2 tea spoons in 250 ml water) is recommended [20, 48, 58, 79]	1	CAMBRA
	Pit and fissure sealants	24. For medium-risk adults, sealant application in caries-prone areas is recommended [17, 33, 55, 80–82]	4	CariesCare
				ICCMS
		25. For high-risk adults, sealant application in caries-prone areas is recommended [17, 33, 55, 80–82]	1	CariesCare
				ICCMS
		26. For extreme-risk adults, sealant application in caries-prone areas is recommended [17, 33, 55, 80–82]	1	CariesCare
				ICCMS
Operative treatment threshold	ICDAS 1,2 occlusal lesions (Incipient caries without dentine involvement or obvious cavity)	27. For low-risk adults, ICDAS 1,2 occlusal lesions (incipient caries without dentine involvement or obvious cavity), if are active, should be managed non-operatively and if are inactive, should be assessed at follow-up sessions for any changes [33, 53–56, 81–85]	1	CariesCare
				CMS
		28. For medium-risk adults, ICDAS 1,2 occlusal lesions if are active, should be managed non-operatively and if are inactive, should be assessed at follow-up sessions for any changes [33, 53–56, 81–85]	1	CariesCare
				CMS
		29. For high-risk adults, ICDAS 1,2 occlusal lesions, if are active, should be managed non-operatively and if are inactive, should be assessed in follow-up sessions for any change [33, 53–56, 81–85]	1	CariesCare
				CMS
		30. For extreme-risk adults, ICDAS 1,2 occlusal lesions, if are active, should be managed non-operatively and if are inactive, should be assessed at follow-up sessions for any changes [33, 53–56, 81–85]	1	CariesCare
				CMS
	ICDAS 3 occlusal lesions (Enamel micro-cavities without an underlying dentin shadow)	31. For low-risk adults, ICDAS 3 occlusal lesions can be managed non-operatively [33, 53–56, 81–85]	1	CariesCare
				CMS
		32. For medium-risk adults, ICDAS 3 occlusal lesions can be managed non-operatively [33, 53–56, 81–85]	1	CariesCare
				CMS
		33. For high-risk adults, ICDAS 3 occlusal lesions can be managed non-operatively [33, 53–56, 81–85]	1	CariesCare
				CMS
		34. For extreme-risk adults, ICDAS 3 occlusal lesions can be managed non-operatively (33, 53–56, 81–85)	4	CariesCare
				CMS
	ICDAS 4 occlusal lesions (Enamel micro-cavities with an underlying dentin shadow)	35. For low-risk adults, ICDAS 4 occlusal lesions can be managed non-operatively, only if the lesion is inactive and the radiolucency does not engage the whole outer third of dentine [33, 53–56, 81–85]	1	CariesCare
				CMS
		36. For medium-risk adults, ICDAS 4 occlusal lesions should be managed operatively [33, 53–56, 81–85]	1	CariesCare
				CMS
		37. For high-risk adults, ICDAS 4 occlusal lesions should be managed operatively [33, 53–56, 81–85]	1	CariesCare
				CMS
		38. For extreme-risk adults, ICDAS 4 occlusal lesions should be managed operatively [33, 53–56, 81–85]	1	CariesCare
				CMS
	C1, C2 proximal lesions (Outer half and inner half of enamel)	39. For low-risk adults, C1, C2 proximal lesions do not need restoration, application of topical fluoride and follow-up is recommended [33, 53–56, 81–85]	1	CariesCare
				CMS
		40. For medium-risk adults, C1, C2 proximal lesions do not need restoration, application of topical fluoride and follow-up is recommended [33, 53–56, 81–85]	1	CariesCare
				CMS
		41. For high-risk adults, C1, C2 proximal lesions do not need restoration, application of topical fluoride and follow-up is recommended [33, 53–56, 81–85]	2	CariesCare
				CMS
		42. For extreme-risk adults, C1, C2 proximal lesions do not need restoration, application of topical fluoride and follow-up is recommended [33, 53–56, 81–85]	2	CariesCare
				CMS
	C3 proximal lesions (Just into dentinoenamel junction)	43. For low-risk adults, C3 proximal lesions do not need restoration, application of topical fluoride and follow-up is recommended [33, 53–56, 81–85]	4	CariesCare
				CMS
		44. For medium-risk adults, C3 proximal lesions do not need restoration, application of topical fluoride and follow-up is recommended [33, 53–56, 81–85]	4	CariesCare
				CMS
		45. For high-risk adults, operative management of C3 proximal lesions is recommended [33, 53–56, 81–85]	1	CariesCare
				CMS
		46. For extreme-risk adults, operative management of C3 proximal lesions is recommended [33, 53–56, 81–85]	1	CariesCare
				CMS
	C4 proximal lesions (Outer third of dentin	47. For low-risk adults, C4 proximal lesions do not need restoration, application of topical fluoride and follow-up is recommended only if the radiolucency does not engage the whole outer third of dentine and there is no cavity after teeth separation. Otherwise, operative management is recommended [33, 53–56, 81–85]	4	CariesCare
				CMS
		48. For medium-risk adults, operative management of C4 proximal lesions is recommended [33, 53–56, 81–85]	1	CariesCare
				CMS
		49. For high-risk adults, operative management of C4 proximal lesions is recommended [33, 53–56, 81–85]	1	CariesCare
				CMS
		50. For extreme-risk adults, operative management of C4 proximal lesions is recommended [33, 53–56, 81–85]	1	CariesCare
				CMS
Follow up	Follow up interval	51. For low-risk adults, 12 months follow-up interval is recommended [20, 48, 57–59, 86]	1	CAMBRA
		52. For medium-risk adults, 6 months follow-up interval is recommended [20, 48, 57–59, 86]	1	Malaysian
				CAMBRA
				CMS
		53. For high-risk adults, 4–6 months follow-up interval is recommended [20, 48, 57–59, 86]	1	CAMBRA
		54. For extreme-risk adults, 3–4 months follow-up interval is recommended [20, 48, 57–59, 86]	1	CAMBRA
				CMS

^1^Priority of each recommendation based on RAND/UCLA appropriateness method:

1 = appropriate, total agreement/ 2 = appropriate, partial agreement/ 3 = uncertain, total agreement/ 4 = uncertain, partial agreement

^2^In other guidelines, NaF mouthrinse was not recommended for this group due to prescription of 5000 ppm F toothpaste

^3^In office NaF gel can be prescribed as a substitute of NaF varnish with similar intervals

NaF = Sodium Fluoride

**Table 4:** Summary of the final clinical recommendations by experts in adapted guideline

Intervention	Caries risk categories^1^			
	Low-risk	Medium-risk	High-risk	Extreme-risk
Fluoride concentration in toothpaste	1000–1450	1000–1450	1450 or more	1450 or more
NaF mouth rinse	−	+	+	+
Chlorhexidine mouthrinse	−	−	+	+
Fluoride gel/ varnish	−	Every 6 months	Every 4–6 months	Every 3–4 months
At home fluoride gel	−	−	−	+
Mouth buffering	−	−	−	+
Pit and fissure sealant	−	+	+	+
*Occlusal caries management*				
ICDAS 1,2	N-OP	N-OP	N-OP	N-OP
ICDAS 3	N-OP	N-OP	N-OP	N-OP
ICDAS 4	N-OP	OP	OP	OP
*Proximal caries management*				
C1,2	N-OP	N-OP	N-OP	N-OP
C3	N-OP	N-OP	OP	OP
C4	N-OP	OP	OP	OP
Follow up intervals	12 months	6 months	4–6 months	3–4 months

^1^Based on caries risk assessment with reduced Cariogram software or CAMBRA tool

NaF = Sodium Fluoride/ N-OP = Non-Operative Treatment/ OP = Operative Treatment

The main preventive and non-preventive measures recommended by expert panels were as follows:

### Fluoride toothpaste and mouthrinse

As for the fluoride concentration in the toothpaste, source guidelines recommended the use of 5000 ppm F toothpaste in high-risk and extreme-risk groups, as it is effective without the need for additional fluoride mouthrinse and hence improves the patient’s compliance [7, 14, 15, 38]. Due to unavailability of 5000 ppm F toothpaste in Iran, the expert panel recommended the use of 1000–1450 ppm F toothpaste in combination with fluoride mouthrinse in medium, high and extreme-risk groups [40, 41]. For high and extreme-risk groups, the 1450 ppm fluoride toothpaste was recommended, which seems reasonable due to its higher effectiveness compared to the 1000–1250 ppm F toothpaste [42]. As a substitute, brushing with F toothpaste more than twice a day was not recommended due to insufficient evidence [43]. Despite the need for standardization and labelling of the local fluoride products, most fluoride toothpastes contain an optimum level of fluoride (1000 ppm) [44, 45].

### Chlorhexidine mouthrinse

The literature is controversial regarding the use of chlorhexidine (CHX) mouthwash for caries prevention. According to a guideline by Anuwar et al. [39] adapted from a Scottish guideline [46], chlorhexidine mouthrinse administration is not recommended due to insufficiency of evidence. This statement is based on a study by Twetman [47] in 2004 that reviewed 22 controlled clinical trial studies on chlorhexidine gel or mouthrinse, and found that only one study compared the effectiveness of CHX mouthrinse with educational program. On the contrary, the CAMBRA guideline [7] recommends the use of chlorhexidine mouthrinse in addition to fluoride mouthrinse based on two randomized clinical trials in 2012 and 2018. In these studies, the use of chlorhexidine mouthrinse for one week every month and fluoride mouthrinse in other weeks, was effective in reducing the load of Streptococcus mutans and caries increment [20, 48]. Similarly, the ICCMS guideline states that “chlorhexidine may be considered as a preventive measure in some countries” [14]. In our guideline, the panel members adopted a similar regimen for high and extreme-risk patients although it might not be accepted by all patients due to its difficulty.

### Fluoride gel/varnish

The expert panel suggested no professionally-applied fluoride for low-risk patients. For other risk groups, fluoride therapy at 3- to 6-month intervals was recommended in line with the latest national guideline on fluoride therapy [49]. The expert panel members preferred fluoride varnish over gel as it is more convenient and less time consuming although its stickiness may be unpleasant for adult patients. In the ADA [35] and ICCMS [14] guidelines, gel or varnish administration is recommended and neither is preferred to the other. However, evidence on fluoride varnish is more conclusive as suggested by a more recent meta-analysis [50]. The main factors to be considered are the high fluoride concentration, i.e., 22,600 ppm in varnish and 12,300 ppm in gel and the regular application [35]. At-home fluoride gel application was recommended for extreme risk groups, in case of caries progression despite using other measures [7].

### Pit and fissure sealants

The ICCMS guideline recommends sealing all at-risk surfaces in medium-risk, high-risk, and extreme-risk patients [14]. However, in the present study, the expert panel members considered this recommendation as uncertain and had incomplete agreement over it in medium-risk patients since it may not be cost-effective to use this method for medium-risk adults in our setting [51]. In addition, the quality of sealant application by some dentists may not be ideal [52].

### Restorative treatment threshold

Regarding the restorative treatment threshold, although there is evidence on the probability of the cessation of proximal caries limited to the enamel, dentinoenamel junction (DEJ), or outer third of dentine [53–55], there were different recommendations on non-restorative treatment of these lesions in guidelines. In this guide, the expert panel members accepted recommendations on non-restorative treatment of proximal caries limited to DEJ in low-risk and medium- risk patients and proximal caries limited to outer third of dentine in low-risk patients with uncertainty and incomplete agreement. Non-restorative treatment of lesions limited to outer third of dentine is recommended in ADA, CMS, ICCMS, and CariesCare guidelines [14, 15, 33, 38]. The expert panel members rejected recommendations on non-restorative treatment of proximal caries limited to outer third of dentine in medium-risk patients with complete agreement in contrast to the CMS guideline [15].

The main concerns of the expert panel regarding non-restorative treatment were non-attendance of patients for routine follow-up in Iran and a high probability of caries progression and pulpal involvement. In a study by Schwendicke et al., the international experts recommended non-restorative treatment for proximal caries limited to outer third of dentine. However, it was argued that the treatment threshold needed to be modified if the patient’s risk modification was unsuccessful or reassessment of the caries risk was not possible [56].

### Follow-up interval

In the adapted guideline, 3- to 12-month risk-based follow-up intervals, consistent with in-office fluoride therapy intervals, were recommended. A recent systematic review indicated no or little difference between 6-month and 3- to 24-month risk-based follow-up intervals during which no specific preventive intervention was provided [57]. However, studies offering preventive care at certain intervals reported decreased caries increments [20, 48, 58, 59]. The panel members did not approve 24-month intervals in low-risk patients due to possible shifts in the caries risk in the long term based on the patient’s condition such as experiencing emotional difficulties, pregnancy, or diet change.

## Discussion

In the present study, after reviewing the relevant CPGs and using the AGREE II tool, 54 recommendations on preventive measures including the use of fluoride toothpaste, fluoride, and chlorhexidine mouthwash, at home and in-office fluoride gel, fluoride varnish, mouth buffering, and sealant, non-preventive measures including restorative treatment threshold, and follow-up interval were adapted. The majority of these recommendations were considered “appropriate” with “total agreement” after a two-round ranking according to the RAND-UCLA appropriateness method by a multidisciplinary expert panel.

In the present study, to ensure reproducibility, appropriate manuals including the AGREE II and RAND-UCLA appropriateness method were used to select high-quality guidelines and achieve consensus. Regarding the AGREE II appraisal checklist, the cut-off points for acceptance were set based on previous similar studies [39, 60]. The RAND-UCLA appropriateness method was preferred to informal consensus or classic Delphi since it offers a more systematic and reproducible approach [61–63].

Our method was similar to the methods used by Lee et al. [60] as the “ADAPTE process and Delphi consensus” and by Irajpour et al. [64] as the “modified ADAPTE”. By contrast, in a study by Anuwar and Ab-Murat [39], the recommendations were finalized by receiving feedback from external reviewers and addressing them in the development committee.

Many guidelines recommended similar preventive measures. However, these measures were distributed differently among risk groups in these guidelines. This matter highlights the great influence of contextual factors, especially financial sources, and disease prevalence on formulating the recommendations [65].

In the present study, we did not include very low-risk groups in the classification, which seems reasonable due to the high prevalence of caries in Iran and the common culture of not attending regular dental check-ups [66]. However, very low-risk groups might be considered in Scandinavian countries as described by Bratthall and Hänsel [9] in the Cariogram tool guide.

To develop a practical guideline, as proposed by the New Zealand Guidelines Group [67], we embraced preventive and non-preventive measures that were considered to be very important and cost-effective according to the literature and experts’ opinions. Moreover, there were practice variations regarding their distributing among different risk groups.

We included preventive measures that are regionally available at a reasonable cost such as fluoride varnishes, which are sufficiently produced by the domestic industry. Products that are not currently available in Iran such as chlorhexidine varnishes or imported products that are available but at high price such as Casein Phosphopeptide-Amorphous Calcium Phosphate (CPP-ACP) pastes and xylitol gums were not included and may be reconsidered in future updates of the adapted guideline. Some other measures that are already well accepted and similar in all guidelines for all risk groups, like “diet counselling” were excluded only from the adaptation process and will be added to final implementation protocol as proposed by the national model for CPG adaptation.

To the best our knowledge, the present study offers the first adapted guideline on caries risk management for Iranian adults. It also distributes different available preventive and non-preventive measures based on the caries risk. We clearly defined risk groups based on validated CRA tools. A specific method or tool for CRA is not proposed in many caries management guidelines, which may act as a possible barrier for the widespread uptake of the guideline as mentioned by external reviewers of a similar study [39].

In the present study, we considered the best available evidence and the opinions of a multidisciplinary expert panel on costs, benefits, adaptability, and acceptability by dentists and patients. The patients' values and preferences were not directly considered and should be further evaluated and addressed during guideline implementation. Another limitation of the present study was that we could not take advantage of key policy-makers or administrative experts of the Ministry of Health in the adaptation process due to time limitations.

As another potential limitation, cost assessment was limited to expert panel members’ discussions on the cost of different products and services in public or private settings, indicating the need for further economic evaluation. It should be noted that this study is the initiation of the ongoing process of CPG adaptation and further stages including external review, publication, implementation, monitoring and periodic updates are still in the planning stages.

## Conclusions

In the present study, an adapted clinical guideline was prepared for risk-based management of dental caries in the adult population. This adapted clinical guideline must help clinicians and healthcare providers to deliver preventive oriented services to improve the oral health and reduce health expenditures in Iran. The next stages of this project are external validation, feasibility assessment, addressing patients’ values and preferences, economic evaluations, and stakeholders’ actions. Writing a proposal for these stages and formal publication of the chairside guideline in national websites are in progress.

## Supplementary Information

**Additional file 1.** Title of data: Search strategies used for identifying further guidelines, recent systematic reviews and local data based on PubMed advance search. Description of data: Describing search strategies that were used for identifying further guidelines, recent systematic reviews and local data based on PubMed advance search.

**Additional file 2.** Title of data: Definitions of measurement of agreement for different panel sizes based on RAND/UCLA Appropriateness Method. Description of data: Describing the method used for assessing agreement between expert panel members.

**Additional file 3.** Title of data: Preliminary screening of relevant CPGs on risk-based caries management. Description of data: Describing title, author, country, year, availability and type of recommendations of relevant CPGs for preliminary screening.

## Data Availability

The datasets used and/or analyzed during the current study are available from the corresponding author on reasonable request.

## Abbreviations

ADA: American Dental Association
ADAPTE: A Manual and Resource Toolkit for guideline adaptation
AGREE II: Appraisal of Guidelines for Research & Evaluation II tool
CAMBRA: Caries Management by Risk Assessment
CMS: Caries Management System
COI: Conflict of interest
CPP-ACP: Casein phosphopeptide-amorphous calcium phosphate
DEJ: Dentinoenamel junction
ICCMS: International Caries Classification and Management System
NICE: National Institute for Health and Care Excellence
PIPOH: Patient population, intervention, professional, outcome, healthcare setting
RAM: RAND/UCLA appropriateness method
SIGN: Scottish Intercollegiate Guidelines Network
