# Supplementary material for: Clinical practice guideline adaptation for risk-based caries management in 18–55 year-old Iranian adults
Source: BMC Oral Health. 2023 Jan 6;23:7. doi: 10.1186/s12903-022-02699-w (PMC9824988; doi:10.1186/s12903-022-02699-w)
Supplement: Supplementary file 1 — Additional file 1. Title of data: Search strategies used for identifying further guidelines, recent systematic reviews and local data based on PubMed advance search. Description of data: Describing search strategies that were used for identifying further guidelines, recent systematic reviews and local data based on PubMed advance search. [file 12903_2022_2699_MOESM1_ESM.pdf]

Additional file 1

Title: Search strategies used for identifying further guidelines, recent systematic reviews and local data based on PubMed advance search.

((((((((Dental Caries [MeSH Terms]) OR (Tooth Demineralization [MeSH Terms])) OR (Dental Care [MeSH Terms])) OR (dentists[MeSH Terms])) OR (Dental Caries Susceptibility [MeSH Terms])) OR (caries management[Title/Abstract])) OR (caries risk[Title/Abstract])) OR (caries risk assessment[Title/Abstract])) OR (caries prevention[Title/Abstract])) OR (preventive dentistry[Title/Abstract]). Filters: Government Publication, Guideline, Practice Guideline, from 2000 – 2022, sorted by the most recent

(((((((((Toothpastes[MeSH Terms]) OR (Sodium Fluoride[MeSH Terms])) OR (Mouthwashes[MeSH Terms])) OR (Dentifrices[MeSH Terms]))) OR (varnish[Title/Abstract])) OR (gel[Title/Abstract])) OR (fluoride therapy[Title/Abstract])) OR (Chlorhexidine[MeSH Terms]))) AND ((((dental caries[MeSH Terms]) OR (Tooth Demineralization[MeSH Terms])) OR (dental care[MeSH Terms])) OR (dentists[MeSH Terms])) Filters: Meta-Analysis, Systematic Review, from 2015 - 2022 Sort by: Most Recent

((((home[Title/Abstract]) OR (home-use[Title/Abstract])) OR (daily[Title/Abstract])) AND ((((gel[Title/Abstract]) OR (foam[Title/Abstract])) OR (tray[Title/Abstract])) OR

(paste[Title/Abstract]))) AND ((fluoride*[Title/Abstract]) OR (NaF[Title/Abstract])) Filters: Meta-Analysis, Systematic Review, from 2015 - 2022 Sort by: Most Recent

(((dental caries[MeSH Terms]) OR (Tooth Demineralization[MeSH Terms]) OR (dental care[MeSH Terms]) OR (dentists[MeSH Terms])) AND ((((((routine[Title/Abstract]) OR (regular[Title/Abstract])) OR (interval*[Title/Abstract])) OR (periodic[Title/Abstract])) OR (frequency[Title/Abstract])) OR (six-month[Title/Abstract]))) AND (((((((check-up*[Title/Abstract]) OR (check up*[Title/Abstract])) OR (recall*[Title/Abstract])) OR (follow-up*[Title/Abstract])) OR (follow up*[Title/Abstract])) OR (visit*[Title/Abstract])) OR (screening*[Title/Abstract])) Filters: Meta-Analysis, Systematic Review, from 2010 - 2022 Sort by: Most Recent

((baking soda[Title/Abstract]) OR (sodium bicarbonate[Title/Abstract])) AND (((((((Mouthwashes[MeSH Terms])) OR (mouth rinse*[Title/Abstract])) OR (mouth-rinse*[Title/Abstract])) OR (mouthwash*[Title/Abstract])) OR (rinsing[Title/Abstract])) OR (mouth wash*[Title/Abstract])) Filters: Meta-Analysis, Randomized Controlled Trial, Systematic Review, from 2015 - 2022 Sort by: Most Recent

((((((Toothpastes[MeSH Terms]) OR (Sodium Fluoride[MeSH Terms]) OR (Mouthwashes[MeSH Terms]) OR (Dentifrices[MeSH Terms]) OR (varnish[Title/Abstract]) OR (gel[Title/Abstract]) OR

(fluoride therapy[Title/Abstract]) OR (Chlorhexidine[MeSH Terms])) OR (non-restorative[Title/Abstract])) OR (non-operative[Title/Abstract])) OR (conservative[Title/Abstract])) OR (((dental caries[MeSH Terms]) OR (Tooth Demineralization[MeSH Terms]) OR (dental care[MeSH Terms]) OR (dentists[MeSH Terms])) OR (preventive dentistry[MeSH Terms]))) AND (((Iran[Title/Abstract]) OR (Iranian[Title/Abstract])) OR (persian[Title/Abstract])) Filters: Clinical Trial, Government Publication, Guideline, Meta-Analysis, Practice Guideline, Randomized Controlled Trial, Systematic Review
