# Supplementary material for: Clinical practice guideline adaptation for risk-based caries management in 18–55 year-old Iranian adults
Source: BMC Oral Health. 2023 Jan 6;23:7. doi: 10.1186/s12903-022-02699-w (PMC9824988; doi:10.1186/s12903-022-02699-w)
Supplement: Supplementary file 2 — Additional file 2. Title of data: Definitions of measurement of agreement for different panel sizes based on RAND/UCLA Appropriateness Method. Description of data: Describing the method used for assessing agreement between expert panel members. [file 12903_2022_2699_MOESM2_ESM.pdf]

Additional file 2

Title: Definitions of measurement of agreement for different panel sizes based on RAND/UCLA Appropriateness Method.

Number of experts	**Disagreement**		**Total agreement**
	Number of experts with scoring in:		Number of experts that their score isn't within the triad range of median:
	Range 7-9	Range 1-3	
8-9-10	3 or more	3 or more	2 or less
11-12-13	4 or more	4 or more	3 or less
14-15-16	5 or more	5 or more	4 or less
