# Supplementary material for: Clinical practice guideline adaptation for risk-based caries management in 18–55 year-old Iranian adults
Source: BMC Oral Health. 2023 Jan 6;23:7. doi: 10.1186/s12903-022-02699-w (PMC9824988; doi:10.1186/s12903-022-02699-w)
Supplement: Supplementary file 3 — Additional file 3. Title of data: Preliminary screening of relevant CPGs on risk-based caries management. Description of data: Describing title, author, country, year, availability and type of recommendations of relevant CPGs for preliminary screening. [file 12903_2022_2699_MOESM3_ESM.pdf]

Additional file 3

Title: Preliminary screening of relevant CPGs on risk-based caries management

No.	Author	Title	Country	Publication year	Availability of full guideline	Providing specific risk-based recommendations for caries risk management
1	Anuwar et al(37)	Developing Clinical Practice Guidelines for Dental Caries Management for the Malaysian Population through the ADAPTE Trans-Contextual Adaptation Process	Malaysia	2021	Yes	Yes
2	Featherstone et al(7)	Evidence-Based Caries Management for All Ages-Practical Guidelines (CAMBRA)	USA	2021	Yes	Yes
3	Hayashi et al(52)	Evidence-based consensus for treating incipient enamel caries in adults by non-invasive methods: recommendations by GRADE guideline	Japan	2020	Yes	No
4	Martignon et al(38)	CariesCare practice guide: consensus on evidence into practice	UK	2019	Yes	Yes
5	Slayton et al(34)	Evidence-based clinical practice guideline on nonrestorative	USA	2018	Yes	No

		treatments for carious lesions				
6	Public Health England(53)	Delivering better oral health: an evidence-based toolkit for prevention	UK	2017	Yes	No
7	World dental federation(54)	Caries Prevention and Management Chairside Guide	Switzerland	2017	No	Yes
8	Association of the Scientific Medical Societies in Germany(55)	Caries prevention in permanent teeth – basic recommendations	Germany	2016	Yes	No
9	National Institute for Health and Care Excellence(56)	Oral health promotion: general dental practice	UK	2015	Yes	No
10	Pitts et al(14)	ICCMS™ Guide for Practitioners and Educators	UK	2014	Yes	Yes
11	Plemons et al(57)	Managing xerostomia and salivary gland hypofunction	USA	2014	Yes	No
12	Weyant et al(36)	Topical fluoride for caries prevention	USA	2013	Yes	Recommendations are exclusively for elevated-risk patients
13	Scottish Dental	Oral Health	Scotland	2012	Yes	No (only providing

	Clinical Effectiveness Programme(58)	Assessment and Review				specific follow up intervals for each risk group)
14	NHS Health Scotland(59)	Oral Health and Nutrition Guidance for Professionals	Scotland	2012	Yes	No
15	Rethman et al(21)	Nonfluoride caries-preventive agents	USA	2011	Yes	No
16	Evans et al(15)	The Caries Management System: an evidence-based preventive strategy for dental practitioners. Application for adults	Australia	2008	Yes	Yes
17	National Institute for Health and Care Excellence(60)	Dental checks: intervals between oral health reviews	UK	2004	Yes	No
